# Psychological Distress Model Among Iranian Pre-Hospital Personnel in Disasters: A Grounded Theory Study

**DOI:** 10.3389/fpsyg.2021.689226

**Published:** 2021-11-10

**Authors:** Maryam Azizi, Abbas Ebadi, Abbas Ostadtaghizadeh, Abbasali Dehghani Tafti, Juliet Roudini, Mohammad Barati, Hamid Reza Khankeh, Reza Bidaki

**Affiliations:** ^1^Faculty of Nursing, AJA University of Medical Sciences, Tehran, Iran; ^2^School of Public Health, Shahid Sadoughi University of Medical Sciences and Health Services, Yazd, Iran; ^3^Behavioral Sciences Research Center, Life Style Institute, Baqiyatallah University of Medical Sciences, Tehran, Iran; ^4^Nursing Faculty, Baqiyatallah University of Medical Sciences, Tehran, Iran; ^5^Department of Disaster and Emergency Health, School of Public Health, Tehran University of Medical Sciences, Tehran, Iran; ^6^Clinical Psychology and Psychotherapy, Department of Psychology, University of Leipzig, Leipzig, Germany; ^7^Infectious Diseases Research Center, AJA University of Medical Sciences, Tehran, Iran; ^8^Health in Emergency and Disaster Research Center, University of Social Welfare and Rehabilitation Sciences, Tehran, Iran; ^9^Research Center of Addiction and Behavioral Sciences, Shahid Sadoughi University of Medical Sciences, Yazd, Iran

**Keywords:** paramedical personnel, disasters, burnout, psychological distress, Iran

## Abstract

**Objective:** Pre-hospital personnels (PHPs) who work in disasters under extreme pressure, uncertainty, and complex situations are victims of disasters themselves, and there is a link between experiencing such incidents and mental health problems. Because most studies focus on the injured and less on the psychological issues of PHPs, the present study aimed to develop a model to provide relief for PHPs in disasters from a psychological perspective.

**Methods:** A grounded theory methodology recommended by Corbin and Strauss (2015) was employed. PHPs (*n* = 24) participated in a semi-structured interview between July 2018 to May 2020.

**Results:** In the analysis of the pre-hospital staff interviews, three main themes were extracted, namely, providing relief with struggle (complexity of incident scenes, command-organizational and occupational challenges), psychological distress (psychological regression and psychological empowerment), and consequences (resilience and job burnout). Seven categories and 22 subcategories were explored from our data *via* the grounded theory approach

**Conclusions:** The PHPs managed psychological distress with two approaches: psychological self-empowerment and regression, which resulted in resilience and burnout, respectively. Due to the lack of enough support, the resilience of the PHPs was short-term, turned into burnout over time, and affected the structural factors again as a cycle.

## Introduction

Pre-hospital personnel (PHP), as front liners in clinical care, encounter thousands of dead and injured people and many destroyed buildings with limited resources in disasters, as a part of their job which makes them hidden victims of disastrous events themselves (Everly et al., 2014; Pourvakhshoori et al., 2017). At the same time, PHP take on roles like planning, responsiveness, prevention, team leadership, triage management, problem-solving, and resource management in complex situations (Xue et al., 2020).

According to the Job Demand Resource Model (JD-R), job demands can lead to negative outcomes like job burnout, activated by an energy depletion process (Weng and Abdullah, 2019). Working in highly stressful environments, with few resources and excessive workload and demands, expose PHPs to psychological distress and other psychological symptoms like dreams, nightmares, feelings of helplessness, numbness, guilt, deprivation, depression, anger, anxiety, frequent recollection of events, changes in social, occupational, and family activities, and other symptoms that can be summarized as post-traumatic stress disorder (PTSD) and job burnout (Alizadeh et al., 2020; Aminizadeh et al., 2020; Delshad et al., 2020).

Psychological problems are more prevalent in the occupation of PHPs than in other stressful jobs (Petrie et al., 2018). In this regard, the recent disasters and emergencies in Iran proved that the psychological aspects of disasters, especially among frontliners who have affected themselves in disasters, need to be focused on seriously (Sadeghi and Ahmadi, 2008). In other words, they suffer from the physical and psychological consequences of their operations at the incident scenes.

Although most psychological symptoms decrease with time, some of them can last for months or even years (Van der Ploeg and Kleber, 2003), which in turn affect the level of productivity, quality of performance, and psychological well-being of a person (Goldstein et al., 2016; Alizadeh et al., 2020). Therefore, the mental health and well-being of paramedics are crucial in providing services to patients and their families. As a result, the psychological well-being and skill preparation of PHPs are some of the prerequisites needed for better performance and to reduce conflicts and tensions in this profession (Bayrami et al., 2017). Also, strengthening the adaptive coping strategies of PHPs can reduce their stress and increase their level of resilience (Jamal et al., 2017).

Reviewing the existing theories and models showed: most are chiefly from developed countries in which their conditions and cultures are entirely different. Moreover, most theories and models tried to focus on providing better service to victims and affected people in disasters by preparing nurses to provide qualified care to victims (Pourvakhshoori et al., 2017). In contrast, the present model emphasizes the mental health of the PHPs so that the issue of caring for the caregivers is highlighted. The process of how PHPs deal with traumatic events is also highlighted, and thus, their psychological consequences are more considered than in previous models (Khankeh et al., 2006; Nakhaei et al., 2012). The vital and capable role of human resources is the most critical factor influencing productivity in disasters, so their mental health, in particular the mental health of PHPs, by which their positions due to special conditions such as working in ambulances and urgent situations are different from the other treatment staffs, is of paramount importance. Still, unfortunately, they are often ignored and are marginalized at the scene of care. For this reason, this study focused mainly on PHPs who worked in disasters, as the other models focused on other health care team members (Blake et al., 2012; Pourvakhshoori et al., 2017).

The term “stress” has served as a valuable exploratory approach that aids researchers to integrate rituals that shed light on the multiple stages of the process that links stressful life events to illness. The psychological tradition focuses on individual perceptions of stress caused by life events based on their assessment of the threats posed and the availability of effective resources to deal with them. Stressful events activate emotional states and in turn trigger behavioral and biological reactions with potential downstream consequences for the client. Therefore, taking the necessary countermeasures allows the patient to correct and prevent pathological consequences (Cohen et al., 2016).

Excessive psychological stress and work-related discomfort are often due to work overload and health emergencies, which makes workers prone to sleep disorders and poor performance (Maniaci et al., 2021).

Due to the complexity of the scene of the disaster, a scientific perspective is needed to make the crisis a solvable and manageable condition. No plan and management for disasters could be better than the assumptions made by the behavior of human beings and groups involved in a situation (Leszczyński et al., 2019). In this regard, the present study was designed to explore the process of providing relief by conducting a qualitative study among Iranian PHPs who have experienced big emergencies and disasters in recent years (COVID-19 pandemic, 2020; Ukrainian plane crash, 2018; Plasco building fire, 2017; Gorgan flood, 2012; and Azerbaijan earthquakes).

## Materials and Methods

### Study Design

An inductive qualitative approach, namely, grounded theory, was used. Grounded theory is used to recognize and narrate the meaning and understanding of human experiences and behavior (Corbin and Strauss, 2015).

### Study Settings

In the present study, the research settings included the Tehran Pre-Hospital Emergency Center, National Prehospital Emergency Center, Golestan province Pre-Hospital Emergency Center (for emergency responders as flood-prone areas), and other relevant prehospital emergency centers in Iran. The agreement of the participants determined the time and location of all interviews. The interviews were recorded into digital audio files (MP3), lasted 45 to 60 min, and were transcribed verbatim using MAXQDA (version10) (Verbi Software, Berlin, Germany). This study was conducted from July 2018 to May 2020. Upon the consent of the interviewees, face-to-face interviews were conducted by the first author at their workplaces. Also, the objectives and reasons for the study were stated to the participants.

### Study Participants and Data Collection

Our sampling followed the grounded theory approach and proceeded in two steps. First, we implemented purposeful sampling to provide maximum diversity in the selection of participants. Second, we used theoretical sampling, a strategy based on the emergence and then saturation of concepts, categories, and subcategories used, to develop a substantive theory (Corbin and Strauss, 1990). This was accomplished by performing interviews with the relevant individuals who could help the research team develop concepts and clarify properties and dimensions. For example, when the concept of command and organizational challenges were extracted, we interviewed three prehospital managers to better understand that concept from the perspective of the commanders. Twenty-four participants were selected through purposive sampling, which consisted of 20 PHPs, three prehospital managers, and one physician who had sufficient experience in the field of disasters or participated in more than three mass casualty incidences. Semi-structured interviewing was used as the primary tool for data collection. This strategy allowed the participants to share sensitive personal experiences in a comfortable atmosphere and provided flexibility for interviewers to pursue exciting inquiry lines before moving on to the next topic. First, the interviewees were asked to disclose their recent experiences in disasters and emergencies. Then, based on these responses, other questions like: “What strategies do you use to overcome the stress of the accident scene?” or “What are the consequences of working in traumatic events?” and other related questions were asked. Each interview was read several times to find the next problems. The authors who had experience in psychological interviews and qualitative research conducted the interviews.

### Ethical Considerations

The ethical committee of the Shahid Sadoughi University of Medical Sciences, Yazd, Iran, approved the proposal of study. All participants provided informed verbal consent to participate. The participants were aware that the conversations would be recorded and that they could request to withdraw from the study at any time and to delete or destroy their taped interviews. The research team had an extensive background in mental health.

### Data Analysis

All interviews were transcribed verbatim from the recordings. Following the recommended grounded theory approach of Corbin and Strauss (2015), a constant comparative analysis with simultaneous data gathering and analysis was performed in three phases: open coding, axial coding, and selective coding. During the open coding phase, the research team members used a shared coding plan, and *in vivo* coding was conducted line by line from the transcribed interviews—codes extracted from words or short phrases used by interviewees that were deemed remarkable. Then the codes were integrated and refined to develop concepts, categories, and subcategories. The next phase, axial coding, was implemented to develop the categories and subcategories and try to discover the relationships between them. To reduce the number of types, they were merged or removed as needed. Finally, the selective coding phase was conducted to integrate all the related categories or to link a core category with other categories; this allowed us to extract and develop the theoretical structure into a conceptual model. Conceptual modeling allowed the researchers to consider an understudied phenomenon systematically (Corbin and Strauss, 2015). Throughout the data analysis process, the research team probed and directed questions and frequently compared the structure of the evidence. All the phases were checked and re-checked several times, following the grounded theory approach recommendations.

### Rigor

The five-step analysis method of Lundman and Graneheim was implicated in the content of the qualitative data analysis. Factors including credibility, dependability, transferability, and confirmability were used to establish the validity and reliability of the data. Constant comparison, active listening, prolonged engagement with the data, immersion in the data, as well as data source and investigator triangulation methods were applied for the acceleration of credibility. We documented and registered a record of our analytic activities for audit trailing and finding dependability establishment. Member-checking and peer-checking techniques were used to ensure the confirmability of the study findings and also to improve the transferability of the study findings. We strived to recruit a sample with maximum variation (Corbin and Strauss, 2015).

## Results

In this study, 24 interviews were conducted. The distribution of the demographic information of the interviews is shown in Table 1.

**Table 1:** Distribution of interviews demographic information.

**Variable**	**Number**	**Percentage**
**Educational status**		
1. Diploma	8	33.33
2. Bachelor's degrees	9	37.5
3. Master's degrees	3	12.5
4. PhD	4	16.67
**Service location**		
Ministers of health	2	8.33
Emergency-based	22	91.67
**Job status**		
Politician	2	8.33
Technicians	15	62.5
Nurses	5	20.84
Physician	2	8.33

**Figure 1:**
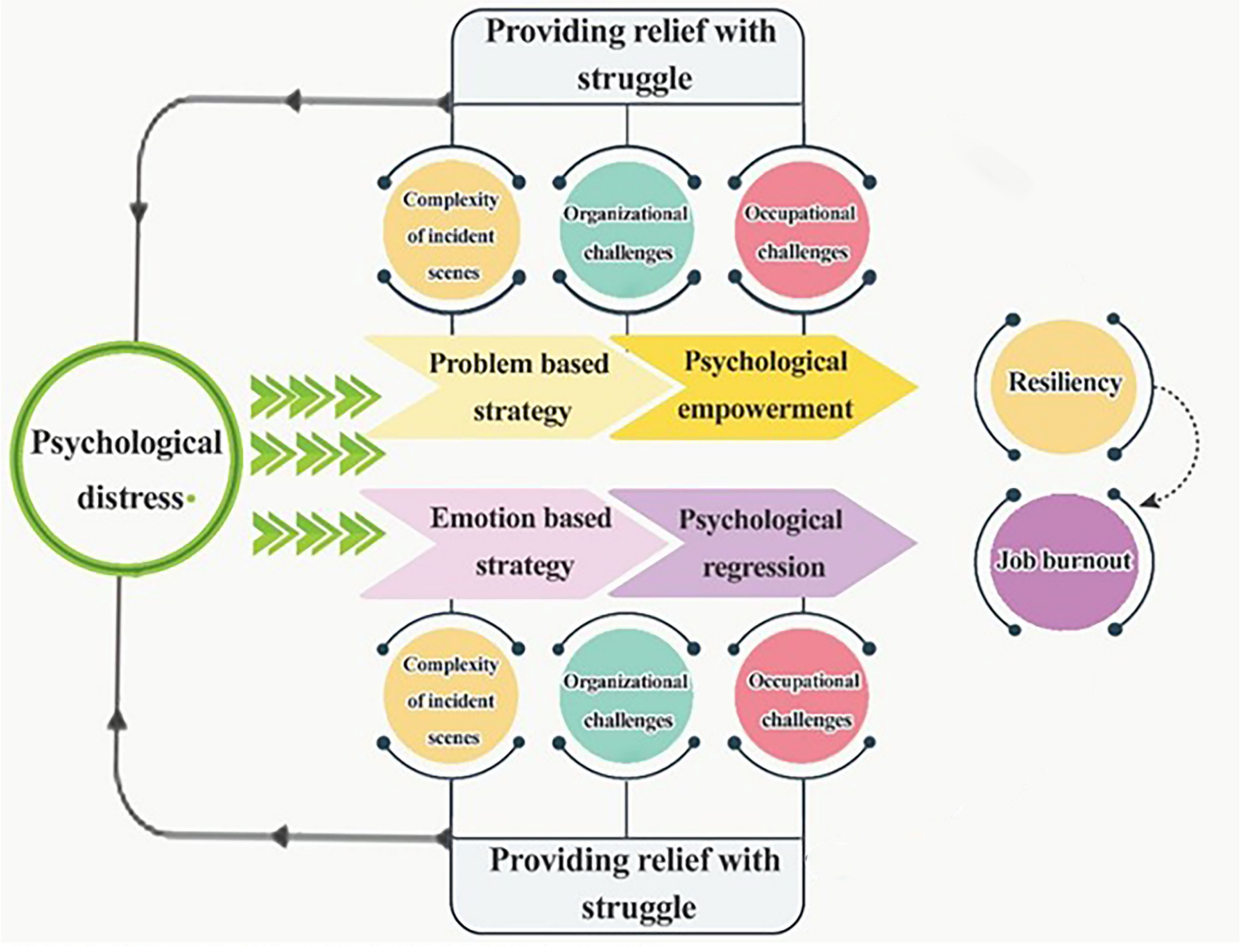
Psychological distress model.

In the analysis of pre-hospital staff interviews, three main themes were extracted, namely, providing relief with struggle, psychological distress, and consequences. Seven categories and 21 subcategories were explored from our data *via* the grounded theory approach (Table 2), enabling us to develop the conceptual model as illustrated in Figure 1. Psychological distress was identified as the core category based on the experiences of the participants in scenes of disaster. Below, we described each of our findings.

**Table 2:** The main category and subcategory.

**Themes**	**Categories**	**Subcategories**
1. Providing relief with struggle:	The complexity of incident scenes	Nature of the incident sceneThreats of the incident sceneSocial demands
	Command-organizational challenges	Shortage of equipment- resourcesPoor organization SupportInadequacy of the commander
	Occupational challenges	Role-related stressorsMoral dilemmasRescuer-related stressors
2. Psychological distress	Psychological regression	Emotional reactionsEmotional behaviorEmotional thoughts
	Psychological empowerment	on-stage strategiesreconstruction
3. Consequences	Resilience	Feeling of identity and efficiencyAcceptanceAltruism
	Job burn out	Emotional-occupational numbnessBehavioral-occupational manifestationsPhysical-occupational erosionpersonal dysfunction

### Providing Relief With the Struggle

Challenges in relief provision are described with the complexity of incident scenes, command and organizational challenges, and occupational challenges which are the PHPs demand in disasters.

The complexity of the incident scene explains the stressors of the scene like the nature and threats of the incident scene and social demands.

The nature of the disaster scene is unique because of the accident extension, chaotic situation, and difficult access to the area of the incident. A participant (p.12), who was part of the first responders in the Mina stampede incident in Mecca in 2015 stated:

“*When we reached the street, we saw a lot of corpses piled on top of each other. I could not imagine this huge number of corpses, but these conditions were very special. I had never witnessed such a huge number of injured people with different degrees of injury and stress.”*

Additionally, insecurity is a major threat to the effective discharge of duties of PHPs which is associated with personal safety, scene safety, and lack of awareness of possible dangers. A participant (p.6), who was a rescue worker in earthquake-affected areas said:

“*In some cases, the companions of the victims abuse us and even to the extent of beating us up. They blame us for the incident and the struggles of their patients. We do not feel safe and secure in front of the patients' companions who have been angered by the plight of their relative victims and who are ready to do any nasty thing.”*

Moreover, in many situations, rescuing done by untrained bystanders, the emotional demands of the people, the failure to recognize the basic welfare of rescuers, and social media demands can lead to many negative social demands.

When asked about rescuing by untrained bystanders, one of the participants (p.3) stated:

“*Sometimes, the bystanders in an attempt to help, endanger both the injured and themselves. In the recent earthquake in Sar-e Pol-e Zahab, a bystander went to help, but he ended up being left under the rubble. Sometimes without knowing the health impact of their actions, they try to drag the victims over without any precautionary measures, which can lead to a spinal cord injury.”*

On the basic welfare and security of rescuers at the incident scene, a participant (p.1) stated:

“*The media has refused to recognize the basic welfare of the rescuer during missions. For example, there was a popular picture of a rescuer who has fallen asleep on the floor during a mission, which went viral on social media. People were angrily commenting that did he go to sleep or to rescue? But they refused to find out the work he had done before getting tired and having some rest There is no welfare security at all and you can't use the facilities during the mission because the media will get on your neck.It is as if the media is waiting to see us in a 'certain' condition during missions so that they can record us and take us to cyberspace and tear us apart.”*

Inadequate human resources, facilities, and equipment denote a shortage of equipment resources. Sometimes the shortage of manpower is due to the inexperience and novice nature of the rescuers. Regarding inadequate human resources, a participant (p.18) said:

“*Among the paramedics, there was a nurse who had a mental problem. He caused us a lot of trouble throughout the mission. To be honest, he was a disaster.”*

A participant (p.12) also commented on the shortage of resources:

“*We were in the Arbaeen mission for about 20 days. The support system was inadequate. We had to search for food under tents for ourselves and sometimes we slept with an empty stomach.”*

Poor organization Support: insufficient emotional, economic, legal, and psychological support describes this component. A participant (p.12), who was part of the Golestan flood rescue team noted:

“*There was no support, no rewards, no incentives, no duty offs. It seems our work was not valued after the mission. Not even a greeting. or even a ceremony was performed to congratulate us. Nothing. There was nothing to keep us going or motivate us for other similar missions.”*

Inadequacy of the commander: Inflexibility, inexperience, lack of coordination, and information problems are the subcategories of the inadequacy of the commander. Participant (p.16), stated:

“*The commander ordered us to go to a flood-hit area to transport a pregnant woman who was in labor with a military truck. The military truck had no medical equipment that helps us in case of emergencies, coupled with severe flooding. We explained to the commander that is it not safe to transport the pregnant women in this vehicle, but he said we just have to obey orders and do as he has ordered.”*

In the occupational challenges category, role-related stressors, moral dilemmas, and rescuer-related stressors explain the challenges related to the prehospital service providing profession.

Role-related stressors: Role conflict, role ambiguity, and role fit (job fit) are the components of role stressors. On this aspect, a participant (p 24) said:

“*You have to be a doctor, technician, or even good psychologist to perform rescuing tasks perfectly. You must know all these very well. If you do not do one well, you and the victim will suffer a lot, you don't know what to do exactly. We do not have defined tasks.”*

Moral dilemmas: Right decisions and unethical requests describe this component. A participant (p.6) said:

“*In the accident scene, many people were injured. Among the victims, there was a mother whose child was in severe discomfort. I had to help her first because of her child's discomfort and I did not follow the principles of triage. I always try to think about which patient I select. Did I make the right decision?”*

A participant (p.8) described unethical requests with this statement:

“*A woman called us and when we got to her house she wanted to give me two kilos of gold to keep for her. This is equivalent to my annual salary, so what should I do now?”*

The rescuer-related stressors include job nature-related stressors, gender vulnerability, loneliness in the scene, family support, and the identification of victims.

Job nature-related stressors were described by a participant (p.3), who was a rescuer in a flood-hit area as:

“*I was on a mission for 40 days in the rainy and cold weather. I did not take off my boots these days. I didn't go home. I had to send my family to another city.”*

On gender vulnerability, a participant (p.3), said:

“*Disgusting scenes have a greater effect on women's morale; also, Field conditions are not suitable for them.”*

A rescuer (p12) who was in the Sar-e Pol-e Zahab earthquake rescue team described loneliness in a scene or arriving earlier with a few personnel in this way:

“*When we experienced the earthquake …, we were few with 1 ambulance in the early hours in the quake-hit areas with lots of injured people and a bunch of rubble. We didn't know what to do all alone.”*

Another participant highlighted the importance of family support, the participant (p.15):

“*I have a husband who is an engineer and has no deep understanding of what the medical profession entails, so he doesn't understand my anxieties.”*

Colleague victims or the identification of the relatives of a colleague as victims was described by a participant (p.21), as:

“*In the Kermanshah earthquake, one of the rescuers' houses was destroyed along with all his family members. I will never forget that scene and the cries of my colleague”*

### Psychological Distress as the Main Problem

In the interviews, one of the main problems of PHPs in emergencies was psychological distress. Following that, two strategies were developed. Some PHPs came up with emotion-based strategies called psychological regression. In contrast, some used problem-based strategies called psychological self-empowerment. These were innovative strategies used at the scene and developed by the rescuers themselves to get rid of the consequences of psychological distress.

If stress overwhelmed the rescuer at the scene of the accident, psychological regression strategies including emotional behavior, emotional reactions, and emotional thoughts explained the reactions of the rescuer when dealing with psychological distress. Extreme emotions, somatization, and shock are the components of emotional reactions. In the case of “extreme reactions,” a participant (p.2) stated:”

“*When I first saw a child drown in flood, I could not speak for a short time. My breath was barely rising.”*

The incident scene sometimes affects the behavior of the PHPs, which includes impaired performance, conflict and challenge, and extreme behavior, which indicates emotional behavior.

“*In the Bam earthquake, after entering the scene and working for several consecutive hours without rest, several rescuers had conflicts together.”*

Emotional thoughts include feelings of helplessness, generalization of events, and catastrophizing. One of the participants (p.2) who was present after the crash of a Ukrainian plane noted:

“*When we arrived at the scene and saw those lots of dead bodies, I felt very helpless when I saw this scene.I mean, I had no power, I was too weak to do anything.”*

Experienced PHPs used problem-based management like supportive, on-stage, and reconstruction strategies when dealing with psychological distress, called psychological empowerment.

Psychological, social, and equipment support are examples of supportive strategies.

A participant (p.12) stated:

“*When we entered the scene and saw the police, firefighters, Red Crescent, and volunteer forces of the people came to help. We were encouraged and less stressed.”*

Another strategy that rescuers used for rational and problem-based management in an accident scene, was on-stage strategies, which included deviation of thought, use of knowledge and experience, and adherence to moral-religious principles. Related statements were said by a participant (p21):

“*In the scene of the accident, we try to place the novice rescuers next to more experienced people to use the knowledge and experience of their colleagues in the scene.”*

Another problem-based management strategy includes reconstruction, composed of psychological arrangements, social support, self-soothing skills, and reassessment that rescuers use after leaving the scene to rebuild their spirits and prepare themselves for the next mission.

In this case, a participant (p2) mentioned:

“*Sometimes when I feel terrible, I talk to myself at the scene of an accident. I tell myself that now I just have to help the patient. If I lose my control, who wants to help them.”*

### Psychological Distress Consequences

As mentioned, after exposing the psychological distress of the scene, the PHPs countered this challenge using two approaches: problem-based and emotion-based approaches. These approaches lead to resilience and job burnout. In the resilience category, we have feelings of identity and efficiency, acceptance, and altruism whereas in job burnout, emotional-occupational numbness, behavioral-occupational manifestations, physical-occupational erosion, and personal dysfunction were obtained. The conceptual model is illustrated in Figure 1.

A participant (p21) stated:

“*After a while, I came to terms with my job and its problems and accepted that I am a relief worker and my job is to serve people in the most difficult situations, and this altruism sense and the feeling of enjoying saving people's lives motivated me to continue.”*

In relation to job burnout, a participant (p11) mentioned:

“*After this period of service in the emergency organization, when I compare myself with the previous one, I see that I became very nervous and quick-tempered. In the family, I do not have the same spirit as before. I became isolated. I just want to retire.”*

## Discussion

The study used the grounded method to develop a substantive theory to clarify the process of providing relief for Iranian PHPs in disasters by identifying several primary related concepts. Based on our findings, the challenges PHPs face include the complexity of the incident scenes (the nature of the disaster scene, chaotic situations, and difficult access), command and organizational challenges (the inadequacy of the commander, equipment resources shortage, and poor organization support) and occupational challenges (the role-related stressors, professional nature-related stressors, rescuers - related stressors, and moral dilemmas). These results are similar to the study of Bayrami, which investigated factors like shortage of human resources and equipment, personnel dissatisfaction, and structural and environmental-social challenges as the most important challenges of the pre-hospital emergency system (Bayrami et al., 2017). In the study of Sorani et al., challenges related to infrastructure, information management systems, employees, management issues, and medical care were raised as the most important challenges for medical service personnel (Yan et al., 2009; Sorani et al., 2018). Additionally, in another study, nine categories of challenges including the structural conditions of prehospital emergency care, medical records, instructions, client-centered therapy, uncertainty about legal consequences, inter psychic challenges, challenges of the emergency team level, feelings of the family caregiver, perception of the illness of the patient, wishes of patients, and social, cultural, and religious contexts of patients and families were identified as the challenges experienced by PHPs (Kamphausen et al., 2019). In addition, other studies were faced with factors like incompetent colleagues, working in unpredictable situations, interference in the provision of care, lack of resources and equipment, barriers to early entry into the scene, forced obedience to the system, poor inter-professional interactions, refusal to care, the challenge of getting satisfaction, and telling the truth as moral distress (Cottrell et al., 2014; Eri et al., 2015; Bayrami et al., 2017; Sorani et al., 2018).

The present study results showed that PHPs experienced two paths while facing these problems: psychological regression, which focuses on emotional coping and leads to burnout, and psychological empowerment, which is similar to problem-oriented coping and leads to resilience. In general, problem-focused coping strategies appear to be more effective in controllable situations, while emotion-focused coping behaviors appear to be more effective in uncontrollable situations. In addition, paying attention to the emotions of people in high-risk situations may interfere with effective decision-making and performance (Delahaij and Van Dam, 2017). In this regard, previous studies showed staff experiences about somatic complaints like gastrointestinal disturbance and some psychiatric symptoms like feelings of hopelessness, impaired interpersonal relationship, hyper aerosol, re-experiencing traumas, and cognitive problems (Raphael et al., 1991; Klappa et al., 2014). Also, the results of different studies showed that anxiety, over-stimulation, hyper-alertness, painful memories, grief, PTSD (Tuckey and Scott, 2014), cognitive impairment, hyperarousal, disruption of interpersonal relationships, loss of interest or social withdrawal, irritability, polydipsia were emotional reactions among the PHPs. Guilt feelings, reconstruction anxiety, irritability, accumulated resentment, and loss of interest in work were some of the symptoms reported in emotional-based coping (Petrie et al., 2018). PHP, regardless of their position or type of organization, have a stressful job that leads to burnout and it is associated with various negative professional and intrapersonal consequences. This syndrome reduces productivity, increases psychological and somatic disturbances, and dissatisfaction with the service (Shareinia et al., 2017). Effective coping mechanisms are positively associated with resilience that enables PHPs to relate to their work challenges. The persons with social performance and good support were more resilient and were able to turn negative experiences into personal development experiences. They are more likely to use positive coping skills such as problem-solving, which leads to resilience (Thompson et al., 2016). Resilience is characterized by the positive adaptation to problems and disadvantages, which mitigate the negative effects of stress and maintain mental balance in stressful work environments (Ebadi et al., 2019).

The personality strengths, appropriate and effective coping strategies, social support, and adequate preparedness of PHPs are also some of the factors that indicate employee resilience (Raphael et al., 1991). Personality traits also affect the vulnerability of the relief forces to stress. A sense of commitment, challenge, and control is a protective personality style for many workers using coping styles that emphasize common problems, constructive use of humor, and use of social support also seem helpful (Ben-Ezra et al., 2013).

The findings identified and examined the interaction between multiple contextual risks, which led to the development of the conceptual model. Accordingly, it is recommended that the mental health status of PHPs should be considered. Safety considerations should be considered in the pre-hospital system. The activation of the Incident Command System (ICS), Emergency Operation Center (EOC), an evaluation system for resource management in the response phase, and organizational support programs like psychological skill workshops, psychological screening, the presence of a counselor in disasters, and follow-up after challenging missions are needed (Sorani et al., 2018). In addition, advanced preparation and standardized protocols could decrease improper interventions in the crash scene.

## Conclusion

This study offered a conceptual model to explain relief is provided for PHPs in disasters. Psychological distress was conceptualized as the main problem with structural factors, psychological empowerment, and regression strategies influencing and reacting to the psychological distress. There were efficient and inefficient consequences from using these strategies. The results held implications for the development of prevention programs. This study, with its in-depth perception of the experiences of the participants, provided a clear picture of their concerns, processes, and responses, and explored the process of providing relief in a disaster. This study, hopefully, would help to produce faster and more effective psychological first aid support to disaster rescuers. Our study identified valuable and effective psychological empowerment skills used by highly resilient PHPs to successfully work in a highly stressful and tension-charged work environment. These strategies could be learned and used for the correction of structural factors and for the comprehensive support toward younger PHPs who reported having less-developed supportive infrastructure around them, to develop target therapies delivered through cognitive behavioral therapy for preventing burnout in PHPs in the future.

## Limitations

One of the limitations of this study was the coordination and the process of obtaining permission to interview prehospital staff, especially in flooded areas. In some cases, we had to interview the staff in the ambulance and during missions. Additionally, interviewing female paramedics was another problem in this study as their number was very limited. It caused the interviewer to plan for several sessions in different places to interview with some of them. Also, the qualitative approach implicated in this study had limitations, and qualitative data cannot be applied to the general population, but it can aid managers in their comprehension, which is an acceptable limitation in all qualitative research.

## Data Availability Statement

The original contributions presented in the study are included in the article/supplementary material, further inquiries can be directed to the corresponding author.

## Ethics Statement

The studies involving human participants were reviewed and approved by Shahid Sadoughi University of Medical Sciences. This article is a part of a doctoral dissertation approved with Ethics Code: IR.SSU.SPH.REC.1400.050. The patients/participants provided their written informed consent to participate in this study.

## Author Contributions

MA, AE, HK, RB, and AO designed the study. MA and MB conducted the interviews and performed the data analysis. RB and AD assisted in the study design and the data analysis. MA, HK, JR, and MB interpreted the data and drafted the manuscript. All the authors read and approved the final manuscript before submission.

## Conflict of Interest

The authors declare that the research was conducted in the absence of any commercial or financial relationships that could be construed as a potential conflict of interest.

## Publisher's Note

All claims expressed in this article are solely those of the authors and do not necessarily represent those of their affiliated organizations, or those of the publisher, the editors and the reviewers. Any product that may be evaluated in this article, or claim that may be made by its manufacturer, is not guaranteed or endorsed by the publisher.

## Abbreviations

PHP: Pre-hospital Personnel.

## References

[B1] Alizadeh A. Khankeh H. R. Barati M. Ahmadi Y. Hadian A. Azizi M. (2020). Psychological distress among Iranian health-care providers exposed to coronavirus disease 2019 (COVID-19): a qualitative study. BMC Psychiatry 20, 1–10. 10.1186/s12888-020-02889-2 33028290 PMC7538532

[B2] Aminizadeh M. Farrokhi M. Ebadi A. Masoumi G. R. Kolivand P. Khankeh H. R. (2020). Hospital preparedness challenges in biological disasters: A qualitative study. Disaster Med. Public Health Prep. 2020, 1–13. 10.1017/dmp.2020.434 33148363 PMC7900656

[B3] Bayrami R. Ebrahimipour H. Hasanzadeh A. (2017). Challenges in Pre hospital emergency medical service in Mashhad: A qualitative study. J. Hosp. 16, 81–89.

[B4] Ben-Ezra M. Palgi Y. Hamama-Raz Y. Soffer Y. Shrira A. (2013). Reactions to the 2011 Tohoku earthquake and tsunami: a preliminary matching study comparing nurses and civilians. J. Nerv. Mental Dis. 201, 534–536. 10.1097/NMD.0b013e318294828e 23719327

[B5] Blake S. Howard D. Eiring H. Tarde S. (2012). San Diego's area coordinator system: a disaster preparedness model for US nursing homes. Disaster Med. Public Health Prep. 6, 424–427. 10.1001/dmp.2012.65 23241474

[B6] Cohen S. Gianaros P. J. Manuck S. B. (2016). A stage model of stress and disease. Perspect. Psychol. Sci. 11, 456–463. 10.1177/1745691616646305 27474134 PMC5647867

[B7] Corbin J. Strauss A. (2015). Basics of Qualitative Research: Techniques and Procedures for Developing Grounded Theory. California, CA: Sage publications.

[B8] Corbin J. M. Strauss A. (1990). Grounded theory research: Procedures, canons, and evaluative criteria. Qual. Sociol. 13, 3–21.

[B9] Cottrell E. K. O'Brien K. Curry M. Meckler G. D. Engle P. P. Jui J.. (2014). Understanding safety in prehospital emergency medical services for children. Prehospital Emerg. Care 18, 350–358. 10.3109/10903127.2013.869640 24669906 PMC4062591

[B10] Delahaij R. Van Dam K. (2017). Coping with acute stress in the military: The influence of coping style, coping self-efficacy and appraisal emotions. Pers. Individ. Dif. 119, 13–18. 10.1016/j.paid.2017.06.021

[B11] Delshad V. Khankeh H. Ebadi A. Bidzan M. Harouni G. Stueck M. (2020). Psychobiological risk assessment in emergency medical service drivers: study protocol for structural equation modeling. Health Psychol. Rep. 8, 453–461. 10.5114/hpr.2020.99455

[B12] Ebadi A. Froutan R. Malekzadeh J. (2019). The design and psychometric evaluation of the emergency medical services resilience scale (EMSRS). Int. Emerg. Nurs. 42, 12–18. 10.1016/j.ienj.2018.09.002 30245058

[B13] Eri M. Jafari N. Kabir M. Mahmoodishan G. Moghassemi M. Tahanian M.. (2015). Concept and challenges of delivering preventive and care services in prehospital emergency medical service: A qualitative study. J. Mazandaran University Med. Sci. 25, 42–57.

[B14] Everly G. S. McCabe O. L. Semon N. L. Thompson C. B. Links J. M. (2014). The development of a model of psychological first aid for non–mental health trained public health personnel: The Johns Hopkins RAPID-PFA. J. Pub. Health Manage. Pract. 20, S24–S29. 10.1097/PHH.0000000000000065 25072485

[B15] Goldstein J. McVey J. Ackroyd-Stolarz S. (2016). The role of emergency medical services in geriatrics: bridging the gap between primary and acute care. Canad. J. Emerg. Med. 18, 54–61. 10.1017/cem.2015.73 26282932

[B16] Jamal Y. Zahra S. T. Yaseen F. Nasreen M. (2017). Coping strategies and hardiness as predictors of stress among rescue workers. Pakistan J. Psychol. Res. 2017, 141–154.

[B17] Kamphausen A. Roese H. Oechsle K. Issleib M. Zöllner C. Bokemeyer C.. (2019). Challenges faced by prehospital emergency physicians providing emergency care to patients with advanced incurable diseases. Emerg. Med. Int. 2019:3456471. 10.1155/2019/3456471 31885924 PMC6899297

[B18] Khankeh H. Mohammadi R. Ahmadi F. (2006). Designing a Model for Health Care Services at the Time of Disaster: A Grounded Theory Study [PhD thesis]: University of Medical Science, Iran.

[B19] Klappa S. Audette J. Do S. J. D. (2014). The roles, barriers and experiences of rehabilitation therapists in disaster relief: post-earthquake Haiti. Disabil. Rehabil. 36, 330–338. 10.3109/09638288.2013.791726 23688294

[B20] Leszczyński P. Panczyk M. Podgórski M. Owczarek K. Gałazkowski R. Mikos M.. (2019). Determinants of occupational burnout among employees of the Emergency Medical Services in Poland. Ann. Agricult. Environ. Med. 26, 114–119. 10.26444/aaem/94294 30922040

[B21] Maniaci A. Ferlito S. Bubbico L. Ledda C. Rapisarda V. Iannella G.. (2021). Comfort Rules for Face Masks Among Healthcare Workers During COVID-19 Spread. Annali di Igiene: Medicina Preventiva e di Comunita. 10.7416/ai.2021.2439 33797548

[B22] Nakhaei M. Khankeh H. Hoseini M. Parsa Yekta Z. Masoumi G. (2012). Exploration of Social Reintegration Process After Disasters: A Grounded Theory Study. University of Social Welfare and Rehabilitation Sciences, Iran.

[B23] Petrie K. Milligan-Saville J. Gayed A. Deady M. Phelps A. Dell L.. (2018). Prevalence of PTSD and common mental disorders amongst ambulance personnel: a systematic review and meta-analysis. Soc. Psychiatry Psychiatr. Epidemiol. 53, 897–909. 10.1007/s00127-018-1539-5 29869691

[B24] Pourvakhshoori N. Norouzi K. Ahmadi F. Hosseini M. Khankeh H. (2017). Nurse in limbo: A qualitative study of nursing in disasters in Iranian context. PLoS ONE 12:e0181314. 10.1371/journal.pone.0181314 28759598 PMC5536275

[B25] Raphael B. Meldrum L. O'Toole B. J. B. B. M. J. (1991). Rescuers' psychological responses to disasters. BMJ: Br. Med. J. 303:1346. 1760594 10.1136/bmj.303.6814.1346 PMC1671643

[B26] Sadeghi N. Ahmadi M. H. (2008). Mental health preparedness for natural disasters in Iran. Nat. Hazards 44, 243–252.

[B27] Shareinia H. Khalilian R. Bloochi Beydokhti T. Javadi H. (2017). Relationship between job satisfaction and burnout among prehospital emergency staff. Q. J. Nurs. Manage. 6, 9–19.

[B28] Sorani M. Tourani S. Khankeh H. R. Panahi S. (2018). Prehospital emergency medical services challenges in disaster; A qualitative study. Emergency 6:e26. 30009228 PMC6036538

[B29] Thompson G. McBride R. B. Hosford C. C. Halaas G. (2016). Resilience among medical students: the role of coping style and social support. Teach. Learn. Med. 28, 174–182. 10.1080/10401334.2016.1146611 27064719

[B30] Tuckey M. R. Scott J. E. (2014). Group critical incident stress debriefing with emergency services personnel: a randomized controlled trial. Anxiety Stress Coping 27, 38–54. 10.1080/10615806.2013.809421 23799773

[B31] Van der Ploeg E. Kleber R. J. (2003). Acute and chronic job stressors among ambulance personnel: predictors of health symptoms. Occup. Environ. Med. 60, i40–i6. 10.1136/oem.60.suppl_1.i40 12782746 PMC1765729

[B32] Weng L. L. Abdullah M. M. (2019). Job demands-Resources model on employee deviance of emergency services personnel: A proposed framework/Weng Leong Lee & Muhammad Madi Abdullah. Gading J. Soc. Sci. 22, 13–20.

[B33] Xue C. L. Shu Y. S. Hayter M. Lee A. (2020). Experiences of nurses involved in natural disaster relief: A meta-synthesis of qualitative literature. J. Clin. Nurs. 29, 4514–4531. 10.1111/jocn.15476 32869888 PMC7756389

[B34] Yan S. Shih Y.-L. J. C. Research O. (2009). Optimal scheduling of emergency roadway repair and subsequent relief distribution. Comp. Operat. Res. 36, 2049–2065. 10.1016/j.cor.2008.07.002

